# Reprogramming Porosity: The Synthetic Evolution of Pore Engineering in Metal–Organic Frameworks

**DOI:** 10.1021/acsmaterialslett.6c00074

**Published:** 2026-03-16

**Authors:** Ankit Mondal, Kelechi Festus, Prapassara Muangsopa, Suman Mandal, Arnab Ghosh, Ahana Sarkar, Qingsheng Wang, Hong-Cai Zhou

**Affiliations:** † Department of Chemistry, 14736 Texas A&M University, College Station, Texas 77843, United States; ‡ Artie McFerrin Department of Chemical Engineering, 14736 Texas A&M University, College Station, Texas 77843, United States

## Abstract

Metal–organic frameworks (MOFs) provide an exceptional platform for pore engineering due to their modularity, crystallinity, and diverse synthetic routes. Traditionally, porosity was controlled by linker length and metal node selection, but recent advances enable active reprogramming of pore environments through synthetic and postsynthetic modifications. This review highlights strategies to tune pore size, geometry, surface chemistry, and flexibility, including linker exchange and insertion, defect engineering, cluster metalation, and covalent or coordinative postsynthetic modifications. It also discusses the growth of functional species within existing pores. Emphasis is placed on mechanistic design rules that define how far such transformations can proceed while preserving long-range order. By unifying these approaches under synthetic pore reprogramming, the review outlines a framework for systematically modifying MOFs beyond their original structures. This enables access to properties and functions unattainable in pristine materials and lays the foundation for designing programmable porous materials with tailored performance.

## Graphical abstract

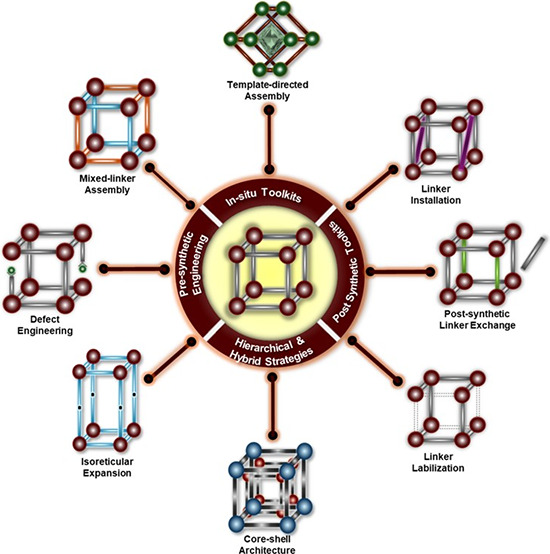

Metal–organic frameworks (MOFs) are unique materials that have been developed to address numerous environmental, medical, technological, and engineering challenges.^1–5^ Since the development of MOF-5 by Omar Yaghi^6^ and his team, this field has seen a boom with many varieties designed for targeted applications. It is a solid-state macromolecular material made from the reaction between a metal node/cluster, which could be metallic clusters or metal ions, and organic linkers/ligands that are usually referred to as ditopic, tritopic, tetratopic, or multitopic, depending on their number of binding sites, which play a significant role in the formation of the MOF and determine its structure and overall properties.^7,8^ It exists in a crystalline form because the bond between the linker/ligand and metal nodes is labile, which makes it reversible and hence can self-correct any defect in the crystal growth process in some cases.^9–11^ This is a unique property because it enables researchers to conduct a fundamental study of their structure and tailor them to specific applications, such as gas capture,^12–15^ catalysis,^16–20^ drug delivery,^21–25^ energy storage,^26–31^ sensing,^32–38^ etc. All these are possible because of pore engineering techniques, which are used to refine their overall size, shape, pores, and chemistry, thereby making them an important and promising component for advancing modern technology.

Pore engineering of MOFs is considered an essential synthetic tool kit by researchers because of the possibility to utilize this strategy to reconstruct, partition, or functionalize them.^39^ It can be done through a *de novo* synthetic approach or postsynthetic modification.^40^ *De novo* synthesis involves the use of carefully and rationally selected starting materials, which include the metal nodes and ligands, solvents, and reaction temperature, to efficiently develop a MOF that is tailored to a specific application in a single reaction step.^11,41–43^ The second approach, postsynthetic modification, is the modification of the structure of an already existing MOF to suit a particular application through its functionalization with moieties that are important for the target application but cannot be incorporated in the first step of the synthesis, thereby necessitating further or multiple-step reaction processes.^44–49^ Although these approaches have been used to develop several MOF materials, they suffer serious setbacks that arise from unwanted or undesirable side reactions that lead to unwanted products, defects, or destruction of sensitive sites.

Linker exchange in MOFs has gained momentum as an alternative to the *de novo* approach as a strategy to overcome its setbacks.^49–52^ It has been used to develop MOFs with several kinds of properties and characteristics for specific applications. This approach is also referred to by some authors as postsynthetic exchange^53–55^ or bridging linker replacement^56–58^ which all imply the same concept as solvent-assisted linker exchange (SALE). Before the invention of this technique, MOFs were considered unmodifiable through a heterogeneous reaction pathway, where mere solvents act as the driving force because of the nature of their structures. Obviously, it would be expected that SALE of shorter linkers will be easy to diffuse into regions that normally will be difficult to get into in the MOF, but practically impossible to achieve for longer chain linkers or ligands. The work of Karagiaridi et al. shows that this is usually not the case, as both short and longer chain linkers can be exchanged in the MOF scaffold successfully through a rational reaction or synthetic pathway.^59–61^ This was shown in their work on SALEM-1, SALEM-2, and SALEM-5, thereby enabling the fine-tuning of the pore volume of these MOFs. It is important to mention that this was an advancement to the work of Burnett et al., who first used the SALE approach to achieve MOF pore volume re-engineering.^58^ Due to the significant benefit of this approach, it has gained attention and has been adopted in the synthesis and redesigning of several MOF materials.

In this review, the synthetic tool kits available to researchers for the synthesis and postsynthetic modification of the pores and structures of MOF materials were explored. This was done systematically, beginning with the fundamental scientific principles guiding pore engineering, to the synthetic toolkits for pore engineering, and finally, the current challenges that still impede the progress of the overall concept of pore engineering in MOF, and what the future direction is to guide researchers in addressing relevant roadblocks through experimentation, computation, and application routes. The optimization and utilization of these techniques will lead to the efficient development of novel MOFs with varied capabilities for multipurpose applications to curb several challenges in the environment and industry.

## 1 Fundamentals of Pore Architecture in MOFs

The defining feature of MOFs is the designed assembly of molecular building blocks into extended, periodic, crystalline pore structures. Unlike the pores of classical porous materials, which arise as a property of the defects in the packing of the constituent building blocks or by phase separation, MOF porosity is encoded into the molecular structure of the material. The study of how pores emerge from the self-assembly of metal-based building blocks and organic linkers, and how their geometry, size, and topology can be controlled, is therefore central to any notion of pore engineering.

### 1.1 Classification of Pore Types

The length scales and connectivities in the pore structures of MOFs are systematically varied and can be classified depending on the size and dimension of their pores. Microporous (pore diameter <2 nm) MOFs having channels and cages such as UiO-66,^62^ ZIF-8,^61^ and HKUST-1,^63^ are highly selective and size-specific due to strong guest–host interactions and are highly efficient gas adsorption and gas separation materials. Elongated or multitopic linker mesoporous (pore diameter 2–50 nm) MOFs, such as MIL-101,^64^ NU-1000,^65^ and PCN-222,^66^ having large pores with voids allow diffusion of large molecules and are effective for confined catalysis and/or molecular component incorporation due to their properties.

Some MOFs display a hierarchical porosity, with micro- and mesopores being present within a single structure. These architectures are obtained through defect engineering, mixed linkers, or template approaches.^67^ The pores of these MOFs can also possess varying dimensionality. One-dimensional channel frameworks, like MOF-74,^68^ show directional transport and severe constraints, whereas three-dimensional cage topologies, such as MIL-101 and PCN-333, show isotropic diffusion in scenarios where there are linkages between the cages.^69^

### 1.2 Pore Metrics and Quantitative Descriptors

For a rational design and comparison among MOFs with respect to their pore structures, certain parameters are adopted on a routine basis. Pore size and diameter are taken to represent the largest sphere that may be accommodated in a cavity or window without straining the support framework. The information on pore size and so on may often be determined from the crystal structure by geometric algorithms, or from the results of gas adsorption studies by density functional theory (DFT) methods of pore size distribution calculations. Pore volume represents the portion of the material that is accessible to guest molecules and correlates directly to storage capacity.

Surface area measurements are usually done via N_2_ adsorption at 77 K and 1 bar. This describes the total internal surface area available for the adsorption process. It has been adopted in the determination and study of record-breaking MOFs such as MOF-210 ^70^ and NU-1500,^71^ whose surface areas are greater than 7000 m^2^/g. This indicates the remarkably large internal surface areas of these materials, which will help in making postsynthetic modifications or target application decisions.

Lastly, the matter of porosity, which is the size and number of pore windows connecting the cavities, is responsible for the transportation and selectivity of molecules. Small pore size can be responsible for kinetic sieving, whereas large pore size can facilitate easy diffusion and exchanges of guests. Moreover, pore connectivity is even more important than pore size in terms of separation and catalytic capabilities.^72–74^

### 1.3 Structural Hierarchy: From Building Units to Pore Systems

Designing MOFs for a specific application relies on the predictable control of their frameworks during synthesis, which is achieved by manipulating three key synthetic determinants, including (i) the connectivity of the metal ions/metal-cluster or secondary building unit (SBU); (ii) the geometry of the organic linker; and (iii) the strategic use of coordination modulation.

The connectivity of inorganic nodes is a primary determinant of the resulting MOF’s topology, including the Zirconium hexanuclear (Zr_6_) oxo cluster, the Cu paddlewheel, and various Co and Fe_3_ clusters. These SBUs possess the versatility in connectivity that enables the targeted synthesis of multiple structures with several topologies.^75^ Among these, Zr-MOFs have attracted much attention due to their versatile connectivity and exceptional stability. As indicated in Figure 1, the first Zr-MOF based on octahedral clusters, UiO-66, with the formula of Zr_6_(μ_3_-O)_4_(μ_3_–OH)_4_(CO_2_)_6_ was reported by Lillerud and co-workers.^62^ This cluster has a 12-connected carboxylate-based cluster and exhibits the face-centered cubic (**fcu**) topology. The high connectivity of the Zr_6_ cluster is a key factor in the remarkable stability of these frameworks. However, the structural possibilities of Zr-MOFs extend far beyond this 12-c SBU. The connectivity of the Zr_6_ clusters can be precisely controlled by employing linkers with different geometries and numbers of coordinating groups, leading to a wide range of structural topologies. For instance, PCN-700 was assembled from an 8-connected Zr_6_O_4_(OH)_8_(H_2_O)_4_ cluster linked with a twisted ditopic ligand, generating a body-centered cubic (**bcu)** topology based on a Zr_6_O_4_(OH)_8_(H_2_O)_4_ cluster.^76^ In another example, PCN-224 was constructed from a 6-connected Zr_6_ cluster and a tetratopic porphyrin ligand (TCPP, tetrakis­(4-carboxyphenyl)­porphyrin).^77^ Topologically, the Zr_6_ cluster can be viewed as a hexagonal node, while the linker can be considered a perfect square. Each Zr_6_ cluster was connected by six adjacent TCPP linkers, creating a unique cubic framework with a (4,6)-connected **she** topology (Figure 1).

**Figure 1:**
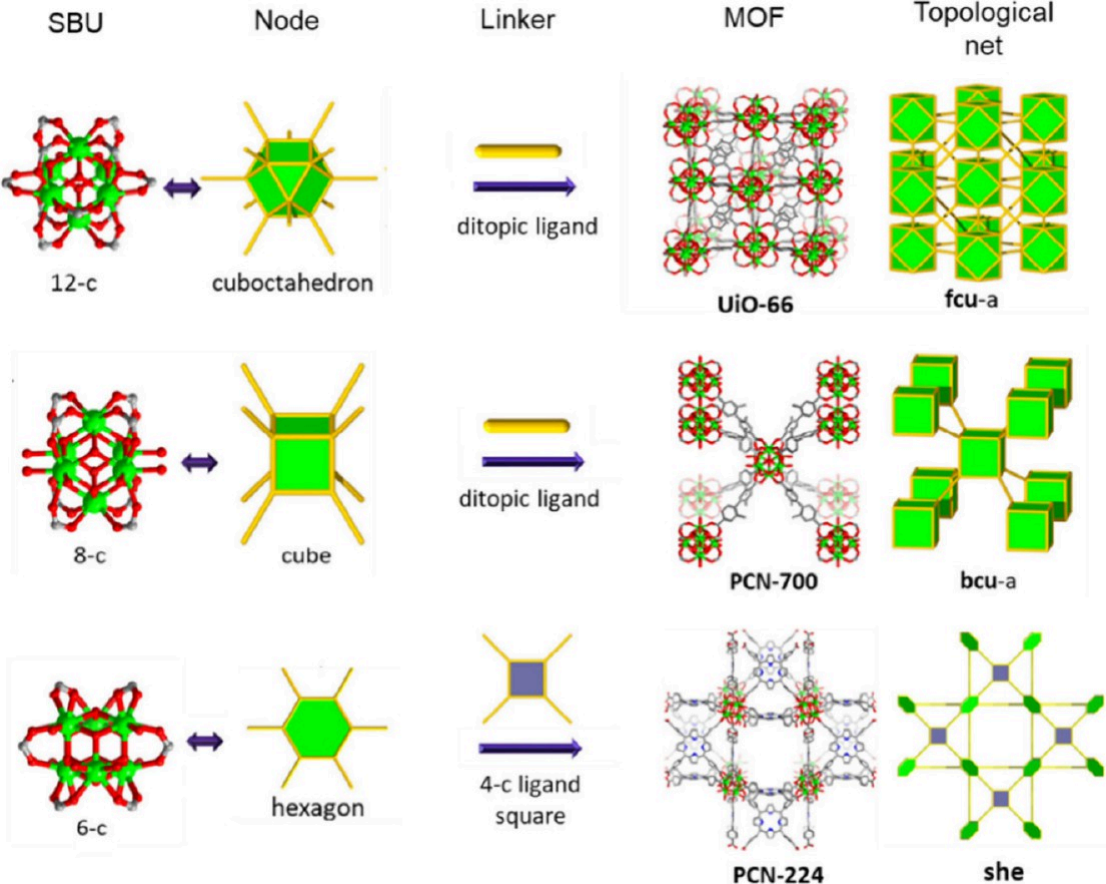
An illustration of representative Zr-MOFs with different 12-, 8-, and 6-connected Zr_6_ nodes. UiO-66,^62^ PCN-700,^76^ PCN-224.^77^ Reproduced with permission from ref 75. Copyright 2019, Elsevier B.V.

The geometry of the organic linker also plays a crucial role in dictating the final structure and topology of MOFs. Connecting SBUs with fixed linking geometries assembles the framework and ultimately determines the structural topology. The progression from simple ditopic linkers to more complex tritopic, tetratopic, or even mixed linkers has enabled the construction of frameworks with increasing connectivity and complexity for a targeted application.^78^ To illustrate this, consider an archetypal MOF-5 (Zn_4_O­(bdc)_3_, bdc = terephthalate), which was synthesized from zinc nitrate [Zn­(NO_3_)_2_] and a ditopic linear bdc linker, producing crystalline MOF-5 under solvothermal conditions (Figure 2).^6^ HKUST-1 is built from the linkage of a planar tritopic 1,3,5-benzenetricarboxylate (btc) linker with a dicopper paddle-wheel SBU.^63^ In this framework, each btc linker connects to three dicopper paddle-wheel SBUs, creating a *T*_d_-octahedron cage (HKUST-1, Figure 2). Six SBUs occupy the vertices of this cage, while four linkers are centered on the alternating triangular faces. The extension of this structure through corner-sharing between adjacent octahedra forms the final cubic framework with a **tbo** topology. Additionally, PCN-222 utilized a tetratopic planar porphyrin ligand (TCPP) as a linker, which connects with 8-connected Zr_6_ clusters to generate a completely different framework with **csq** topology (PCN-222, Figure 2).^66^ These examples demonstrated how increasing the linker topology from two to four fundamentally alters the geometric outcome and the resulting network architecture.

**Figure 2:**
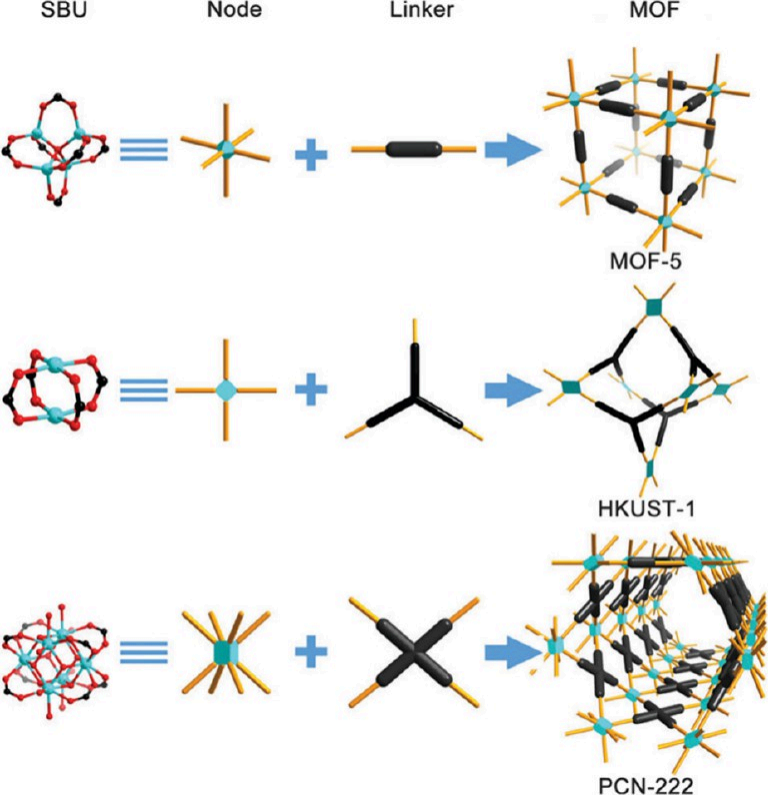
Schematic diagram of the construction of some representative coordination MOFs from different SBUs and linker geometries. MOF-5,^6^ HKUST-1,^63^ PCN-222.^66^ Reproduced with permission from ref 78. Copyright 2014, The Royal Society of Chemistry.

### 1.4 Coordination Modulation

Coordination modulation has emerged as a powerful approach for fine-tuning the properties of MOFs, as first introduced by Kitagawa and co-workers.^79^ This technique commonly applies an auxiliary ligand called a modulator during the MOF synthesis,^80^ in which this modulator can compete with the linker for coordination to the metal ion during the framework assembly, leading to specific morphologies, crystal sizes, and porosity features. A wide variety of chemical species can function as modulators include monodentate carboxylic acids (e.g., formic acid, acetic acid, benzoic acid) just to mention a few,^81^ which are frequently employed to obtain highly crystalline products due to the slowed growth rate of the crystal;^82^ basic modulators (e.g., pyridine, ammonium hydroxide), which increase linker solubility via deprotonation; surfactant modulators (e.g., sodium dodecyl sulfate (SDS)), can either undergo adsorption on MOF surface or function as capping agents to control the size and morphology during the formation process. Therefore, the successful application of this technique on MOF synthesis requires thoughtful consideration of the modulator’s nature, including functional group, p*K*a, and relative concentration. For instance, the choice of modulator can be used to selectively control crystal morphology, as illustrated in the work reported by Kitagawa and co-workers.^83^ The use of acetic acid as a modulator in the synthesis of [Cu_2_(ndc)_2_(dabco)]_n_ MOF (ndc = 1,4-naphthalene dicarboxylate; dabco = 1,4-diazabicyclo[2.2.2]­octane) has been selectively observed in the nanorod structure, as the acetic acid selectively impedes the Cu-ndc coordination. This approach was further developed by modulating the synthesis of this MOF with pyridine and acetic acid or with only pyridine.^84^ When solely pyridine was utilized, its N atom donor capped only the [001] direction, resulting in the formation of a nanosheet. In contrast, the use of both pyridine and acetic acid effectively capped crystal growth in all directions, leading to reduced particle size and the formation of nanocubes (Figure 3).

**Figure 3:**
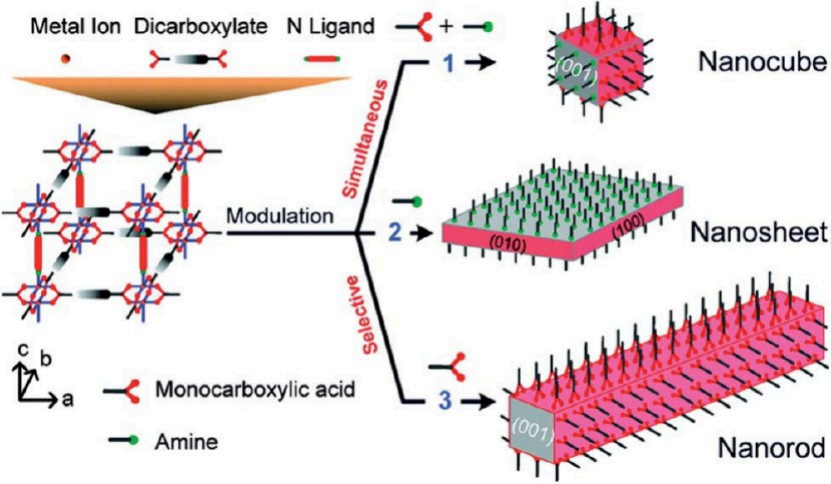
Schematic of coordination modulations on morphologies control of [Cu_2_(ndc)_2_(dabco)]_n_ MOF using simultaneously different modulators. Reproduced with permission from ref 84. Copyright 2012, American Chemical Society.

### 1.5 Concept of Active Pore Environment

Engineering the active pore environment within MOFs enables precise control of the material’s properties for specific application requirements. This optimization is typically achieved by installing specific functional groups,^85–87^ modulating hydrophobicity,^88–92^ introducing pore charge,^93–95^ and designing frameworks with controlled structural flexibility.^96–99^

A primary strategy for creating an active pore environment is the direct installation of specific functional groups on the pore interior, enabling intermolecular interactions between organic linkers and guest molecules. Yaghi and co-workers pioneered a mixed-linker strategy to introduce functionality (functional groups) into MOFs.^85^ They successfully synthesized a multivariate (MTV) MOF-5 crystal from up to eight different linkers in a one-pot reaction. These MOFs were obtained by using linkers of identical length and coordination modes, which enabled functional diversity while preserving the parent framework structure (Figure 4a). Zhou and co-workers developed, for the first time after this work, a highly stable Zr-based MOF with tunable azide loading within the pores. These azide groups then serve as anchors to attach various functional groups via the click reaction. This precise functionalization creates tailored pore environments capable of selective CO_2_ adsorption with amine as the functional group.^86^

**Figure 4:**
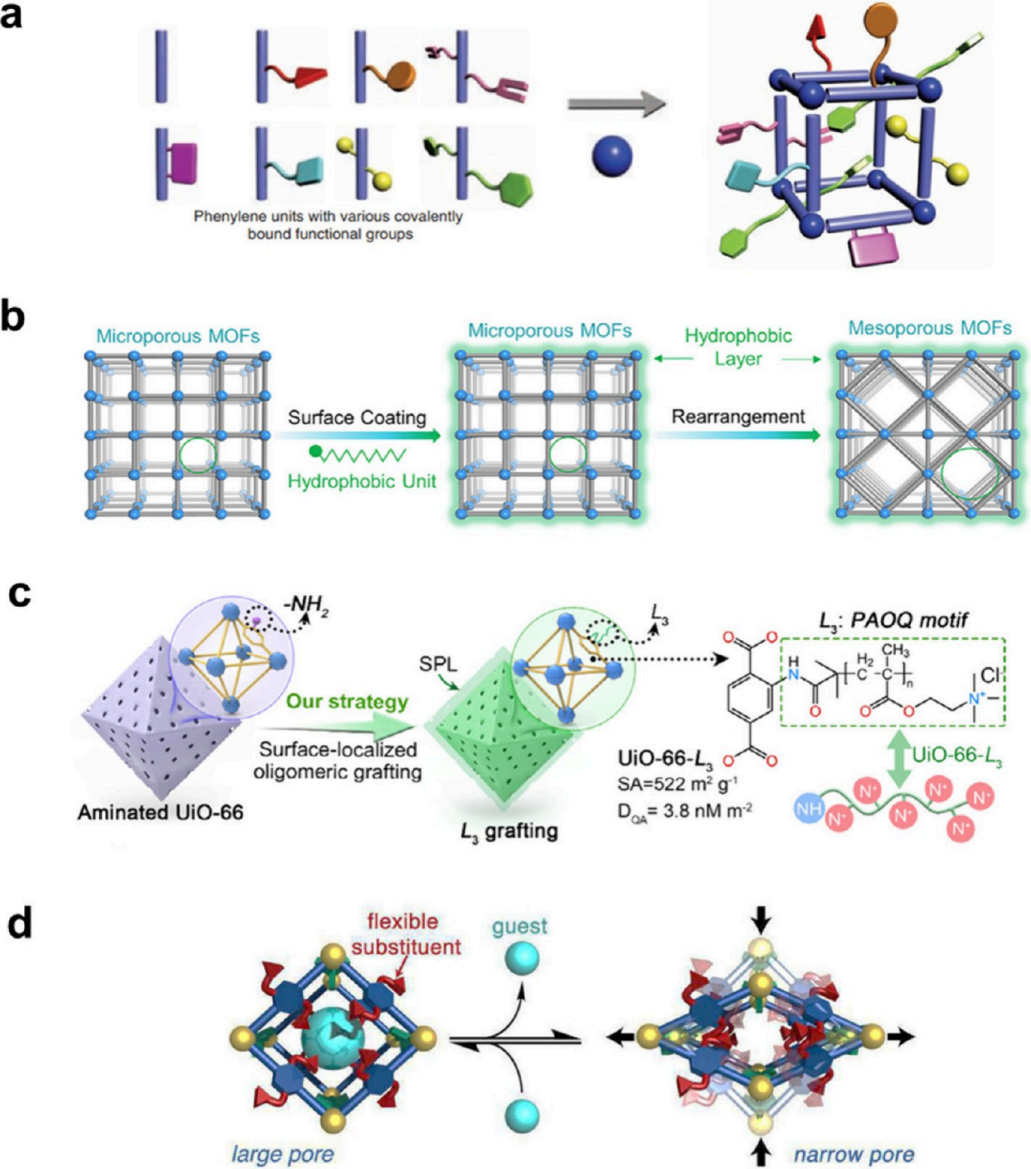
(a) Illustration of the MTV-MOF-5 structure with eight different functionalities on linking groups. Reproduced with permission from ref 85. Copyright 2010, American Association for the Advancement of Science. (b) Superhydrophobic and mesoporous AlTz-68 MOF using the bioinspired encapsulation rearrangement strategy. Reproduced with permission from ref 91. Copyright 2020, Cell Press. (c) Surface grafted UiO-66-L_3_ with a cationic framework for selective PFAS adsorption. Reproduced with permission from ref 95. Copyright 2025, Wiley-VCH. (d) Guest-induced transition of a flexible MOF upon adsorption (large-pore) and guest removal (narrow-pore). Reproduced with permission from ref 96. Copyright 2012, American Chemical Society.

Engineering the pore surface to be hydrophobic is a critical strategy for improving the stability of MOFs in aqueous or humid conditions, particularly for oil/water separation, organic pollutant enrichment, and protection of water-sensitive heterogeneous catalysts.^100–102^ Hydrophobicity in MOFs is commonly introduced through the use of alkyl- or fluorine-containing linkers, either by direct synthesis or postsynthetic modulations.^88,89^ In 2006, Yaghi and co-workers reported on the water resistance of ZIF-8 by introducing methyl groups on the linkers to block water molecules from attacking the [ZnN_4_] units.^92^ The Cohen group introduced long alkyl substituents into IRMOFs to construct hydrophilic pores, resulting in high moisture stability.^90^ However, these methods can lead to pore blockage by the bulky functional groups, which reduces the accessible surface area and limits the scope of their applications. To overcome this, Zhou’s group presented AlTz-68 MOF with intrinsic high hydrophobicity and excellent stability, while maintaining high surface areas and large and uniform pores, via a bioinspired encapsulation rearrangement strategy (Figure 4b).^91^

Furthermore, another approach to pore engineering is to introduce charges within the pores of MOFs. This approach creates electrostatic interactions between the charged backbone of the framework and counterions in the pore, and it is widely useful across various applications. For instance, Yaghi and co-workers reported an intrinsically anionic framework, MOF-688, that enables rapid lithium-ion transport, resulting in high ionic conductivity and making it a promising solid-state electrolyte.^94^ Another critical application is the adsorption of per- and polyfluoroalkyl substances (PFAS). Yu and co-workers reported the cationic MOF UiO-66-L3 by functionalizing a Zr-MOF (UiO-66-NH2) with proximally grafted oligoalkyl-quaternary ammonium (PAOQ) motifs (Figure 4c). This creates a positively charged framework that strongly attracts anionic PFAS molecules, enabling rapid and selective removal.^95^

One last approach is designing flexible MOFs, a concept pioneered by Kitagawa and co-workers as soft porous crystals.^103^ These frameworks change their pore volume in response to external physical and chemical stimuli, such as mechanical stress, adsorption/desorption of guest molecules, temperature, and light, making them suitable for potential applications.^97,98^ For instance, Fisher and co-workers demonstrated that the Zn-based MOF can undergo guest-induced structural transitions by introducing functionalized linkers with flexible side chains.^96^ The resulting MOFs displayed a reversible structural transition upon guest removal and re-expanding again in the adsorption of DMF (*N*,*N*-dimethylformamide), EtOH, or CO_2_ (Figure 4d). Zhou group also reported flexible Zr-based MOFs named PCN-700 series through postsynthetic linker installation. Upon catalytic activity, the resulting flexible Zr-MOFs showed reversible expansion when the catalyst was on, while shrinking when the catalyst was off.^99^

## 2 Synthetic Toolkits for Pore Engineering

Porosity in MOFs is no longer engineered through an initial framework designed to achieve specifically tailored porosity goals but through an array of advanced synthesis techniques to design and redesign pores systematically ordered within the MOF materials. These strategy sets function over an array of scales in space and time, from before synthesis to tailor node to linker size and shape, to after synthesis to introduce chemistry. Now, we categorize these strategy sets in this section according to complementary “presynthetic”, “in situ (assembly stage)”, “postsynthetic” MOF pore engineering capabilities to emphasize how they offer orthogonal routes to MOF pore environment design in increasing detail and complexity as needed.

### 2.1 Synthetic Toolkits for Pre-synthetic Engineering

The presynthetic engineering of MOFs is a concept that refers to the various approaches adopted for their design, without postsynthetic engineering. These are usually used to develop MOFs for targeted applications without the need for further modification or reaction steps. In this section, attention will be given to the roles of Isorecticular expansion,^104–107^ Mixed-linker assembly,^108–110^ coordination modulation,^111–114^ defect engineering,^115–118^ and topological design and retrosynthetic analysis^4,119–122^ in MOF synthesis as pore, size, and shape engineering techniques. These techniques have been extensively reported in the literature and are hence well established but still require further studies and optimization for adoption in industrial large-scale processes.

#### 2.1.1 Isoreticular Expansion

This is a technique that is frequently used in the expansion of MOF pores. It is made possible by the labile bonds between the linker or ligand and metal nodes/clusters, which enable the insertion of a longer ligand in the place of the shorter counterpart for the expansion of the pores of the MOFs. It has been used to expand the pores of MOFs that are considered inert for better optimization and specific applications. He, Tao et al. reported the synthesis of BUT-32 and BUT-33, Nickel­(II) pyrazolate MOFs through this technique from a Ni_3_(BTP)_2_ (BTP^3–^ = 1,3,5-tris­(pyrazolate-4-yl)­benzene) MOF with a **the-a** topological net.^123^ This was achieved through the replacement of the H_3_BTP linker with an elongated version, H_3_TPTA (TPTA^3–^ = 2,4,6-tris­(4-(pyrazolate-4-yl)­phenyl)-1,3,5-triazine). It was observed that BUT-32 is microporous, thermodynamically stable, and interpenetrated in moderate reaction conditions, while BUT-33 is mesoporous, kinetically driven, and does not have interpenetration under vigorous reaction conditions. Although their topology is isoreticular, the microporosity of BUT-33 makes it suitable for catalysis due to efficient mass transfer. Xinyu Yang et al. replaced the BPDC (BPDC = biphenyl-4,4′-dicarboxylate) linker in UiO-67 with AZDC (AZDC = azobenzene dicarboxylate) and NDC (NDC = naphthalene dicarboxylate) (AZDC being the elongated version) linkers and achieved an expansion of the pore window with the AZDC linker.^124^ This modification, coupled with the PCN-222 core, enhanced its capability to function as a size-selective catalyst for olefin epoxidation. In another work, Feng et al. modified the structure of a noninterpenetrated MOF, PCN-160, to PCN-161 through the replacement of the linkers with a labile ligand that has hydrolyzable imine bonds, which was then partially hydrolyzed to induce defects that could find relevance in catalysis and gas capture.^125^ Kim et al. used a series of short to long isoreticular ligands (NDC, BPDC, and AZDC) to generate Zr-NDC, Zr-BPDC, and Zr-AZDC moiré MOFs, which increased the intercluster distance from 1.7 to 2.1 nm, hence are useful in sensing, catalysis, and photonics.^107^ O’Shaughnessy et al. reported the successful synthesis of isoreticular MOFs from trigonal amine linkers (TAPT = 1,3,5-tris­(4-aminophenyl)­benzene), TT = 4,4′,4″-(1,3,5-triazine-2,4,6-triyl)­tris­[benzenamine], and TTBT = 4′,4‴,4⁗′-(1,3,5-triazine-2,4,6-triyl)­tris­[[1,1′-biphenyl]–4-amine]) with varying arm lengths and ammonium halide salts. The TTBT linker had the highest arm length and hence the highest pore size and pore volume.^126^ Additionally, a series of isoreticular AgC-MOFs was designed by Jiang et al. with alkynyl ligands (L1 = 1,3,5-tris­(4-ethynylphenyl)­benzene, L2 = 1,3,5-tris­((4-ethynylphenyl)­ethynyl)­benzene, L3 = 4,4⁗-diethynyl-5″-(4′-ethynyl-[1,1′-biphenyl]-4-yl)-1,1′:4′,1″:3″,1‴:4‴,1⁗-quinquephenyl) of varying lengths and Ag­(CH_3_CN)_3_BF_4_ metal node to form AgC-MOF-1, AgC-MOF-2, and AgC-MOF-3 with permanent pores and water stability.^34^ Just as important, AgC-MOF-3 has the highest pore size because of the length of the linker used to synthesize it.

#### 2.1.2 Mixed-linker Assembly

Mixed linker assembly has been an important synthetic tool kit in the design of certain MOF materials over the years which has contributed significantly to the advancement of the field.^108,110,127–129^ It is especially because the organic linkers are unique building blocks in MOFs, and hence the ability to assemble them rationally in the framework for efficient pore size engineering facilitates MOFs’ applications in many areas of human endeavors. Gao et al. integrated tetrakis (4-carboxyphenyl) porphyrin (TCPP) ligand and a series of linkers such as 1,4-benzenedicarboxylate, biphenyl-4,4′-dicarboxylic acid, and 1,3,5-tri­(4-carboxyphenyl) benzene (H_3_BTB) into Zr-MOFs in a one-pot mixed linker synthetic assembly. The structure of the MOFs was preserved, which enabled the rigidity of the framework and boosted its capability for singlet oxygen (^1^O_2_) generation under irradiation.^130^ Tan et al. developed a mixed linker In-MOF-MF from the assembly of terephthalic acid and fumaric acid.^110^ It was observed that In^3+^ ions have a rigid interaction with both linkers to generate robust nanofibers with good aspect ratios. Overall, this facilitated the fabrication of a novel one-channel p–n junction nanofiber. The gas adsorption capacity of MOF-801-M-FA (M = Zr or Hf) was optimized due to the mixed linker assembly of fumarate and formate ligands by Gu et al.^131^ Under ambient conditions, the IAST (Ideal Adsorbed Solution Theory) selectivity increased significantly for CH_4_/N_2_ and CO_2_/N_2_ mixtures, which makes the MOFs suitable for CH_4_ and CO_2_ capture, as confirmed by the breakthrough experiment studies. Because the main contaminant in coal bed methane (CBM) is Nitrogen, as reported by Ma et al.,^132^ this makes them also suitable for CH_4_ capture for energy applications. Zhou et al. used MOF Eu-FDA, which comprises an Eu-based chain and pentacyclic 2,5-furandicarboxylic acid (H_2_FDA) as a foundation for the design of luminescence sensing MOFs through a mixed ligand approach.^32^ The MOFs developed through this approach were isostructural and achieved through the replacement of the pentacyclic ligand with derivatives of hexacyclic isophthalic acid.

#### 2.1.3 Defect Engineering

Coordination modulation is a synthetic tool kit for defect engineering in MOF materials that has been adopted as a pore engineering technique because of the importance that defects play, such as enhancing gas adsorption capability, photocatalytic properties, and sensing, just to mention a few. This is achieved by controlling the crystal growth process by introducing species that compete with the ligands in the process for coordination to the metal nodes/clusters. Jeffrey Long and his team used a noncoordinating buffer, 4-morpholinepropanesulfonic acid (MOPs), to develop Co_2_(dobdc) (dobdc^4–^ = 2,5-dioxido-1,4-benzenedicarboxylate) from cobalt­(II) acetate, which generated a compressed crystal at the *c*-axis not observed in the traditionally synthesized counterpart without a noncoordinating buffer (MOPs).^133^ This happened because the acetate competed for coordination with the dobdc^4–^ linker in the framework during the synthesis. Ce-MOF-801–50eq is a MOF reported by Zhou et al.^134^ It demonstrated significant enhancement in its catalytic capability for DCPD hydrogenation with a 100% conversion rate because of the defect present in the framework through defect engineering. This is because of the unsaturated metal sites, surface area, and pore size distribution. Fei et al. synthesized a NUC-105a MOF, which acts as a bifunctional catalyst due to the high unsaturated Lewis acidic thulium­(III) center in the framework.^135^ These defects also make NUC-105a behave as a heterogeneous catalyst for CO_2_ cycloaddition and epoxides, which is important industrially.

#### 2.1.4 Topological Design and Retrosynthetic Analysis

Topological design and retrosynthesis have gained relevance in MOF synthesis due to the need to design MOFs with certain kinds of topology and specific attention to geometry matching and linker-to-node connectivity. This is important in the optimization of the shape, pore, stability, and potency of the MOF material. It serves as a solution to random synthesis and aids in the search and design of MOFs for specific applications. Retrosynthetic Analysis is the opposite of the MOF synthesis, which involves a reverse of the process to recover the linker and metal precursors used and subsequently or potentially reassemble them into different and unique designs. Zhou et al. used a topology-guided synthetic approach to synthesize PCN-228, PCN-229, and PCN-230, with **ftw-a** topology and mesoporous pores.^136^ Due to the porphyrinic linkers in these MOFs, they certainly, in addition to their reported stability in aqueous solutions, also possess catalytic properties. Yaun et al. adopted the retrosynthesis approach to reengineer the cavity of a mesoporous MOF through the insertion of metal clusters and organic linkers to develop a bimetallic catalytic system.^137^ Han et al. designed MOFs (LNU-H1, LNU-H2, and LNU-H3) with unique C_2_H_2_/CO_2_/C_2_H_4_ ternary gas separation capabilities and **jcq** topology by using topology blockers (F^–^, Cl^–^, and Br^–^) in addition to six-connected nodes hexa-pyridyl ligands and five-connected undercoordinated Cu^2+^ to redesign the traditional **soc** topology.^138^

### 2.2 In Situ (Assembly-Stage) Toolkits

In situ pore engineering would thus describe those methods in which the morphology, environment, and porous networks of MOFs would be designed and encoded at the initial stages of their synthesis through their crystal growth process itself. In this design strategy, instead of modifying an existing lattice, as in postsynthetic modifications, leverage can be taken from the binding and solvation equilibria available in the system.^139–141^

#### 2.2.1 Template-Directed Assembly

Template-directed synthetic methodologies have been employed to fabricate a diverse range of porous materials and nanomaterials for an extended period (Figure 5).^142^ Weak interactions, including van der Waals forces, hydrogen bonds^143^ and electrostatic forces, between the template guest molecules and MOF precursors dictate the self-assembly process, leading to the formation of certain building blocks that ultimately constitute the final MOF structure. This technology is primarily employed for three principal reasons: (1) to create MOFs that cannot be directly synthesized due to the highly stable metal–ligand interactions that hinder defect correction during crystal development^144^ (2) to manufacture metal–organic frameworks with novel and diverse topologies^145,146^ (3) to include novel functionalities into MOFs utilizing the preserved templates within the cavities.^147,148^

**Figure 5:**
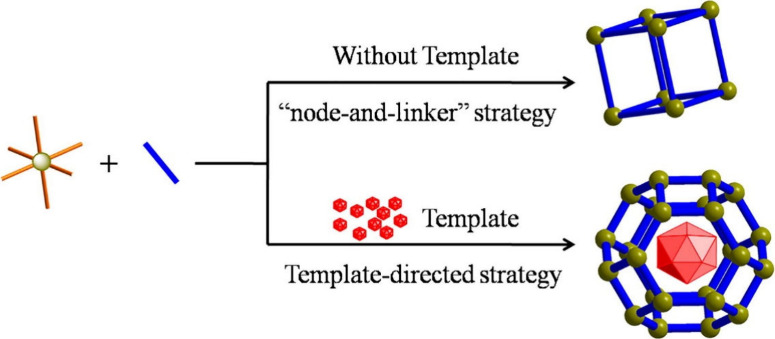
Graphic illustration of the construction of MOFs through the “node-and-linker” strategy or template-directed strategy. Reproduced with permission from ref 145. Copyright 2019, Elsevier.

Representative template-directed strategies illustrate how different types of templates can address the challenges outlined above. To overcome the limitations in direct synthesis, Wei et al. prepared a Cu-based MOF by trans-metalating a Zn-MOF template.^149^ The labile Zn–O coordination bonds promote ligand exchange and dynamic defect repair during growth, allowing the framework to develop high crystallinity before Zn is replaced by Cu. By contrast, direct synthesis of the Cu-MOF typically yields only microcrystalline powders, because the more kinetically inert Cu–O bonds hinder defect repair and restrict crystal development (Figure 6a**)**. A similar example was recently reported by Gómez-Oliveira et al., wherein a metal-induced dynamic topological transformation facilitated the interchange of Ca^2+^ with Co^2+^, resulting in the conversion of MUV-30 into the ultraporous MUV-301 framework, which is inaccessible by direct routes.^150^

**Figure 6:**
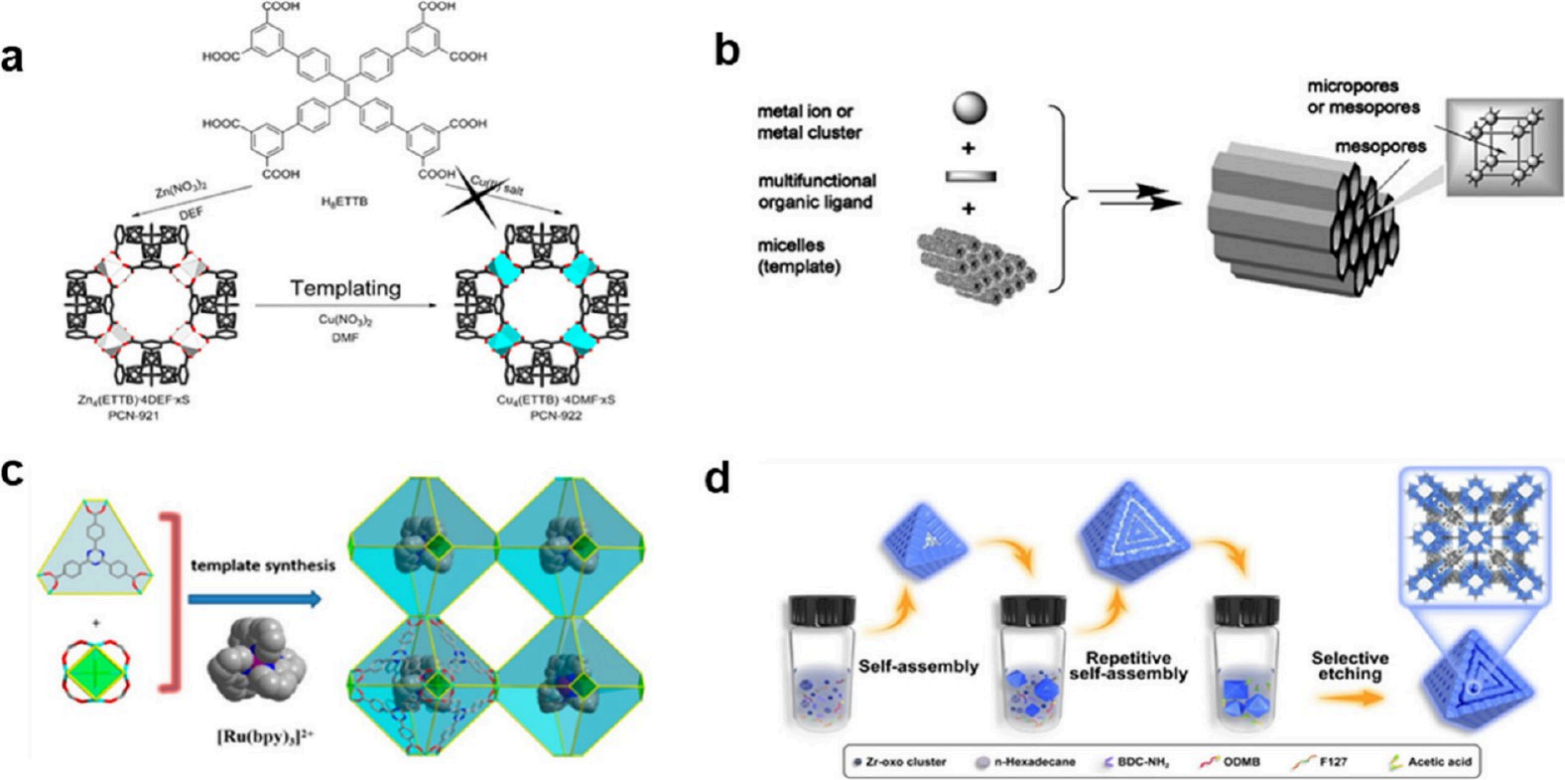
(a) Synthesis of Cu-MOF using Zn-MOF as a template. Reproduced with permission from ref 144. Copyright 2013, American Chemical Society. (b) Mesostructured metal–organic frameworks (MOFs) self-assembled from metal ions and multifunctional organic linkers utilizing micelles as supramolecular templates. Reproduced with permission from ref 151. Copyright 2008, Wiley-VCH. (c) Preparation of photocatalyst-encapsulating MOFs utilizing [Ru­(bpy)_3_]^2+^ complex as a template. Reproduced with permission from ref 153. Copyright 2019, American Chemical Society. (d) Schematic illustration of the dual-template-directed strategy of synthesis of multishell hollow mesoporous UiO-66-NH_2_. Reproduced with permission from ref 154. CC BY 4.0..

Soft surfactant templates have been exploited to engineer hierarchical porosity. Qiu et al., utilized supramolecular aggregates of the surfactant cetyltrimethylammonium bromide (CTAB) as templates to synthesize the very first example of hierarchically micro- and mesoporous MOFs employing Cu^2+^ and benzene-1,3,5-tricarboxylate (btc^3–^) ions as framework-building constituents with tunable porosity (Figure 6b).^151^ Another similar example of using surfactant as templates to create mesopores was given by Zhou et al., where CTAB and a chelating agent (citric acid) were used as a cooperative template system, for the generation of a mesoMOF containing a hierarchical system of mesopores interconnected with micropores.^152^ Functional molecular templates add yet another dimension, as demonstrated by the incorporation of the photoactive [Ru­(bpy)_3_]^2+^ complex as a template to synthesize MOFs, where the template preserves photochemical properties and exploits MOF robustness and porosity, resulting in hybrid photocatalysts with superior heterogeneous catalytic activity and highlighting the value of template-guided MOF synthesis for advanced functional materials (Figure 6c).^153^ A dual-template-directed strategy was reported by Guan et al., where octadecyl dimethyl betaine and polymer surfactant F127 were employed as cooperative templates to direct the self-assembly of multishell hollow mesoporous UiO-66-NH_2_ with tunable shell parameters.^154^ This approach facilitates the creation of complex hierarchical architectures via selective etching, resulting in structures with enhanced catalytic performance and mass transport (Figure 6d). Overall, these examples demonstrate that template-directed strategies provide a powerful and versatile toolbox for accessing otherwise unattainable MOF architectures, hierarchical porosity, and integrated functions.

#### 2.2.2 Solvent/Coordination Environment Tuning

A meticulous choice of reaction solvent is essential, as each solvent can significantly affect the coordination interactions between metal centers and ligands, which frequently results in the formation of distinct framework structures.^155,156^ The coordinating solvents act as structural directing agents,^155,157^ whereas the noncoordinating solvents get incorporated into the structure of MOFs^158^ and act as space-filling molecules.

Chen et al. synthesized four distinct Mn­(II) coordination polymers with the rigid ligand 2,3,5,6-tetrachloro-1,4-benzenedicarboxylic acid (H_2_BDCCl_4_) from different solvent systems, demonstrating that the solvent significantly influences the μ_2_/μ_4_ bridging modes of BDCCl_4_ and therefore the dimensionality of the resultant frameworks (Figure 7a).^159^ Ghosh and Kitagawa provided another instance of a solvent functioning as a structural-directing agent, reporting the synthesis of three novel three-dimensional (3D) coordination polymers through the self-assembly of a flexible tricarboxylate ligand and Cd­(II) metal ion in dimethylformamide (DMF), diethylformamide (DEF), and isopropanol (iPrOH). Variations in solvent size and shape resulted in the formation of solvent-templated neutral and anionic frameworks with distinct pore sizes and configurations.^160^ Alcohol solvents have also been found to play an important role in regulating the final MOF structure, as reported by Cai et al.^156^ In their Ba­(II)-MOF system, subtle changes in the alcohol–water or DMF-alcohol solvent composition, although not leading to solvent incorporation in the final crystal lattices, were sufficient to direct the crystallization into four distinct 3D architectures with different topologies. A related example of solvent-controlled structural transformation involves a 1D chain of interdigitated nanocages, in which discrete cages are linked via Cu–O bonds and bulky hydrophobic groups.^161^ By tuning the hydrophobicity/hydrophilicity of the solvent mixture, a reversible transformation between discrete nanocages and the 1D chain is achieved (Figure 7b). This solvent-responsive process, analogous to micelle formation and protein folding, enhances both the stability and gas-adsorption capacity of the 1D chain relative to the discrete cages.

**Figure 7:**
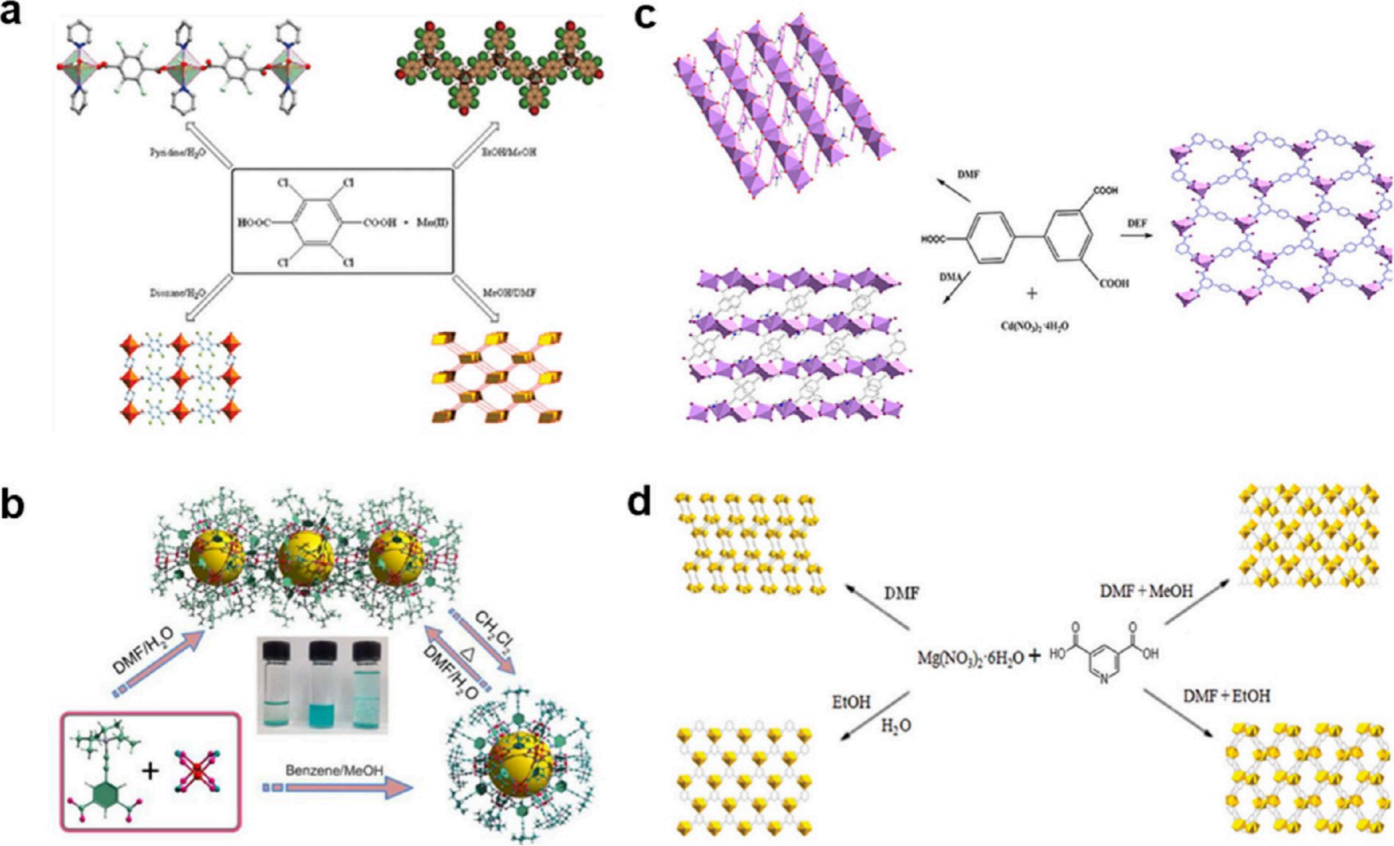
(a) Representation of different solvent systems yielding different morphologies of Mn (II)-H_2_BDCCl_4_ MOF. Reproduced with permission from ref 159. Copyright 2008, American Chemical Society. (b) Representations of the self-assembly mechanisms and transformation of cuboctahedral cages and the one-dimensional chain. Reproduced with permission from ref 161. Copyright 2012, American Chemical Society. (c) Representation of different solvent systems yielding different morphologies of Cd-BPT MOF. Reproduced with permission from ref 163. Copyright 2012, American Chemical Society. (d) Different solvent systems yield different topologies of Mg-MOFs. Reproduced with permission from ref 164. Copyright 2011, American Chemical Society.

In the Co (II) coordination polymers built from H_3_cpbda built by Huang et al. in distinct solvents like DMF, DMA, and DMP, the framework remained the same, although due to the incorporation of the solvents in the MOF, the pore size varied according to the size of the MOFs.^162^ This demonstrates that solvent choice can be used as a simple structural handle to tune channel dimensions in porous coordination polymers by varying the size and coordination behavior of the solvent molecules incorporated into the framework. On the other hand, in some other reports, incorporation of solvents in the MOF’s framework altered the structure of the MOFs totally. Li et al. reported three solvent-dependent Cd­(II) coordination architectures, which were obtained from H_3_BPT and Cd­(NO_3_)_2_ in H_2_O/DMF, H_2_O/DMA, and H_2_O/DEF, respectively.^163^ DMF bridges neighboring Cd centers to give a 3D Cd–O–Cd chain network, whereas the DMA ligand terminally coordinates to a single Cd­(II), and for DEF, a 2D (6,3) honeycomb [Cd­(BPT)] layer stacks via π–π interactions into a 3D supramolecular framework (Figure 7c). The coordination affinity of the solvent plays a major role in determining the final topology of MOFs, as demonstrated by Banerjee and co-workers.^164^ In their study, they established that Mg^2+^ exhibits the highest coordination affinity for water, followed by DMF, whereas methanol and ethanol do not coordinate in the presence of the former solvents. This competitive coordination directed the formation of four distinct Mg-based MOF structures from varying solvent compositions (Figure 7d).

### 2.3 Post-Synthetic Toolkits

Postsynthetic pore engineering is considered to include methods that reprogram the MOF pores through chemical or structural means after the formation of the MOF lattice. In distinction to methods used during the assembly phase, these are applied to an existing lattice to allow for variability in pore size, structure, surface chemistry, and function after maintaining long-range order. Such methods have led to MOFs becoming modular or addressable materials.

Among the most useful postsynthetic strategies is the so-called postsynthetic linker/node modification. Reactive functional groups integrated into the structure, which may be amines, hydroxyl groups, or azides, for example, can be modified through covalent chemical reactions, thus endowing the structure with steric, polar, or catalytic properties. The advantage offered by these materials lies in the ability of covalent chemical reactions, which occur in a rigid structure, to precisely position functional groups in space.

#### 2.3.1 Post-Synthetic Linker Exchange

Postsynthetic linker exchange enables direct reprogramming of MOF pore environments by replacing native linkers while preserving crystallinity and topology. The extent of exchange is governed by framework robustness, linker acidity/basicity, sterics, and mass transport; partial exchange is particularly valuable for creating multivariate or spatially heterogeneous pore surfaces. The first definitive demonstration of this concept was provided by Takaishi et al., who used solvent-assisted linker exchange (SALE) to replace dipyridyl-porphyrin pillars in robust porphyrinic MOFs (RPMs) with chemically distinct metalloporphyrin struts (M-dipy, M = 2H^+^, Al^3+^, Sn^4+^), producing crystalline solid-solution frameworks Zn­(Zn_1–*x*_M_*x*_)-RPM that were inaccessible by direct solvothermal synthesis.^165^ Single-crystal to single-crystal anisotropic linker redistribution along the accessible pores of the materials was confirmed by spatially resolved EDX mapping to follow a diffusion-controlled reaction mechanism rather than the dissolution and recrystallization mechanism of the original material.

Building on this foundation, Karagiaridi et al. demonstrated that SALE can be used not only to modify pore chemistry but also to systematically expand pore dimensions by inserting longer dipyridyl pillars into a pillared-paddlewheel MOF (SALEM-5), generating daughter frameworks (SALEM-6 to -8) with progressively elongated unit cells and enlarged cages while preserving topology.^60^ (Figure 8) The results of mixed-linker studies disclosed that metal–ligand affinity correlation to the original metal centers dictates the enthalpy of the linker redistribution reaction, so that even the introduction of relatively bulkier linkers becomes feasible if they exhibit an advantageous affinity to the metal centers of the structure of the original material.

**Figure 8:**
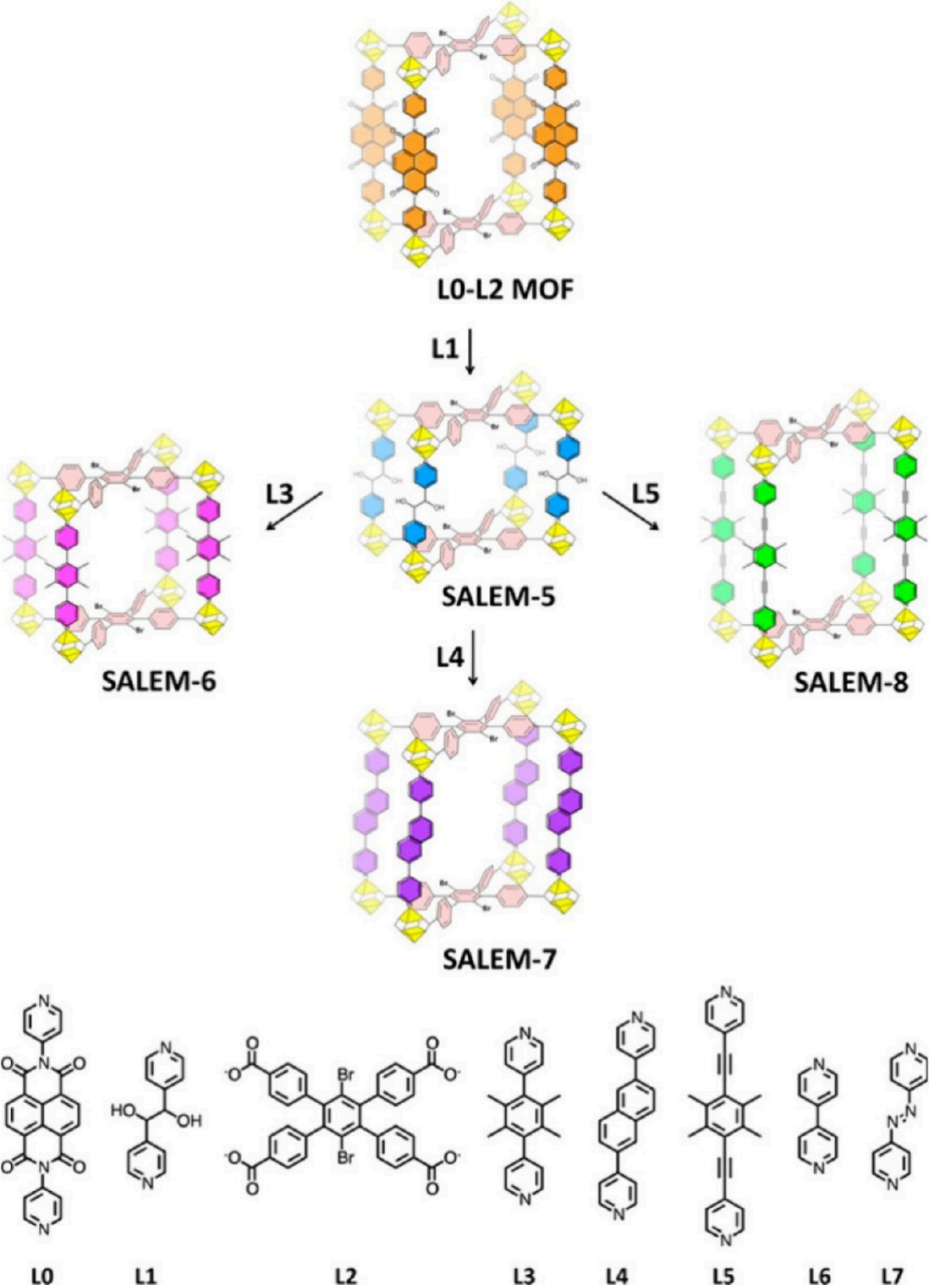
Stepwise solvent-assisted linker exchange (SALE) starting from the parent L0-L2MOF using different organic linkers (L1–L7) to generate an isoreticular series of SALEM frameworks (SALEM-5 to SALEM-8). Reproduced with permission from ref 60. Copyright 2013, American Chemical Society.

This capability is particularly powerful for gas separation and storage, where minute changes in linker length or functionality can precisely tune pore apertures, adsorption sites, and selectivity. In catalysis, linker exchange enables the installation of catalytically active ligands, such as metalloporphyrins, organometallic complexes, or Lewis acidic sites, directly into the pore walls, creating single-site heterogeneous catalysts with programmable activity and spatial arrangement. For adsorption and molecular capture, exchanged linkers can provide polar, hydrogen-bonding, or π-interacting groups, enhancing the framework’s CO_2_, hydrocarbon, or contaminant adsorption capabilities. In confined-space chemistry, nanoparticles and multivariate pore environments, linker exchange enables the design of tailored host lattices and functionalities not available in the parent framework. Linker exchange is central to fulfilling the potential structural tunability that MOFs offer for alloying performance gains in separations, catalysis, and molecular storage.

#### 2.3.2 Linker Installation

A postsynthetic pore modification technique called linker installation is where di or multitopic linkers are inserted into targeted coordination holes of an existing MOF crystal lattice instead of replacing it altogether, like in SALE. The idea was proven using PCN-700, which is a Zr-MOF that contains open and accessible Zr_6_ clusters that allow bridges to be formed by replacing their ending −OH/H_2_O ligands (Figure 9a). In the first-ever example of what came to be known as “sequential linker installation (SLI)”, Yuan et al. found that dicarboxylate linear linkers of varying sizes and functionality could be selectively installed on PCN-700 in a single crystal-to-single crystal manner, where the location of another added dicarboxylate link was directly analyzed and confirmed by SCXRD analysis-a technique that allows functional molecules to be placed at precise locations within its pores.^76^

**Figure 9:**
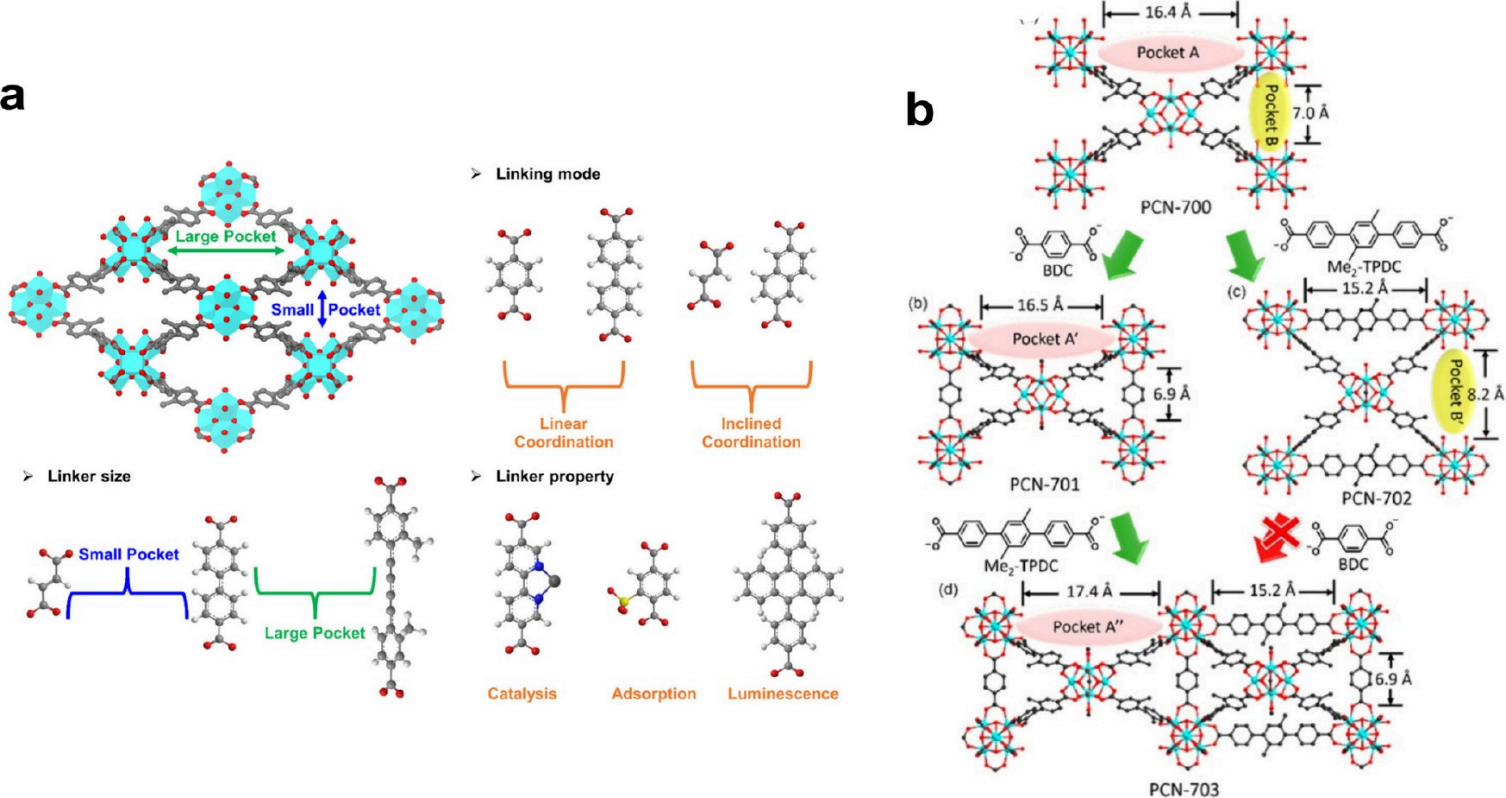
(a) Illustration of PCN-700 highlighting the coexistence of large and small pockets and linker selections for sequential linker installation on PCN-700. Reproduced with permission from ref 166. CC BY 4.0. (b) Sequential linker installation based on PCN-700. Reproduced with permission from ref 76. Copyright 2015, American Chemical Society.

This footing has been extended by a more comprehensive study by Yuan et al. to develop the linker installation method into a more universal toolkit for the design of environments around the pores with carefully positioned functionalities around the structure.^87^ Using the installation of linkers with differing lengths/substituent groups to the platforms of the type PCN-700, they devised series of derived materials where the volume of the pores, the constraints imposed by the pores’ apertures, and the chemical microenvironments could differ while preserving the crystalline materials in essence, transforming the “defects” with missing linkers/under-coordinated nodes into functionalized docking regions for pore site “rewriting”. More recent research endeavors have further extended the methodology to the stepwise assembly of high multivariate frameworks of MOFs with high order (e.g., quinary) where multiple differing linkers could also be step-wisely installed to provide an atomistic design strategy to environments of cooperative adsorption and catalysis that would be more difficult to implement with the use of the conventional one-pot method involving the installation of mix-linkers sufficient for the synthesis of the desired materials. A current outlook would incorporate these achievements into the conceptual rubric “sequential linker installation” (Figure 9b).

More recently, Deng, Xiong and co-workers showed that dynamic linker modification allows the parent MOF, LIFM-28 to be transformed into a sequential multifunctional platform for converting CO_2_ through the sequential introduction of catalytic linkers.^167^ A series of derived frameworks, LIFM-DSL-3, -6, and -8, prepared through stepwise modification with amine, NHC-CO_2_, bpy-Co, and sulfonated linkers, selectively promoted the hydrosilylation reaction, *N*-methylation, cyclic carbonate, and heterocycle formation from CO_2_, respectively. In each reaction, the introduced linker not only determined the active site and the local pore environment, thereby improving CO_2_ binding and stabilizing reaction intermediates to achieve high reactivity and selectivity under mild conditions, but also paved the way for further modification to address the challenge of efficiently utilizing CO_2_ (Figure 10).

**Figure 10:**
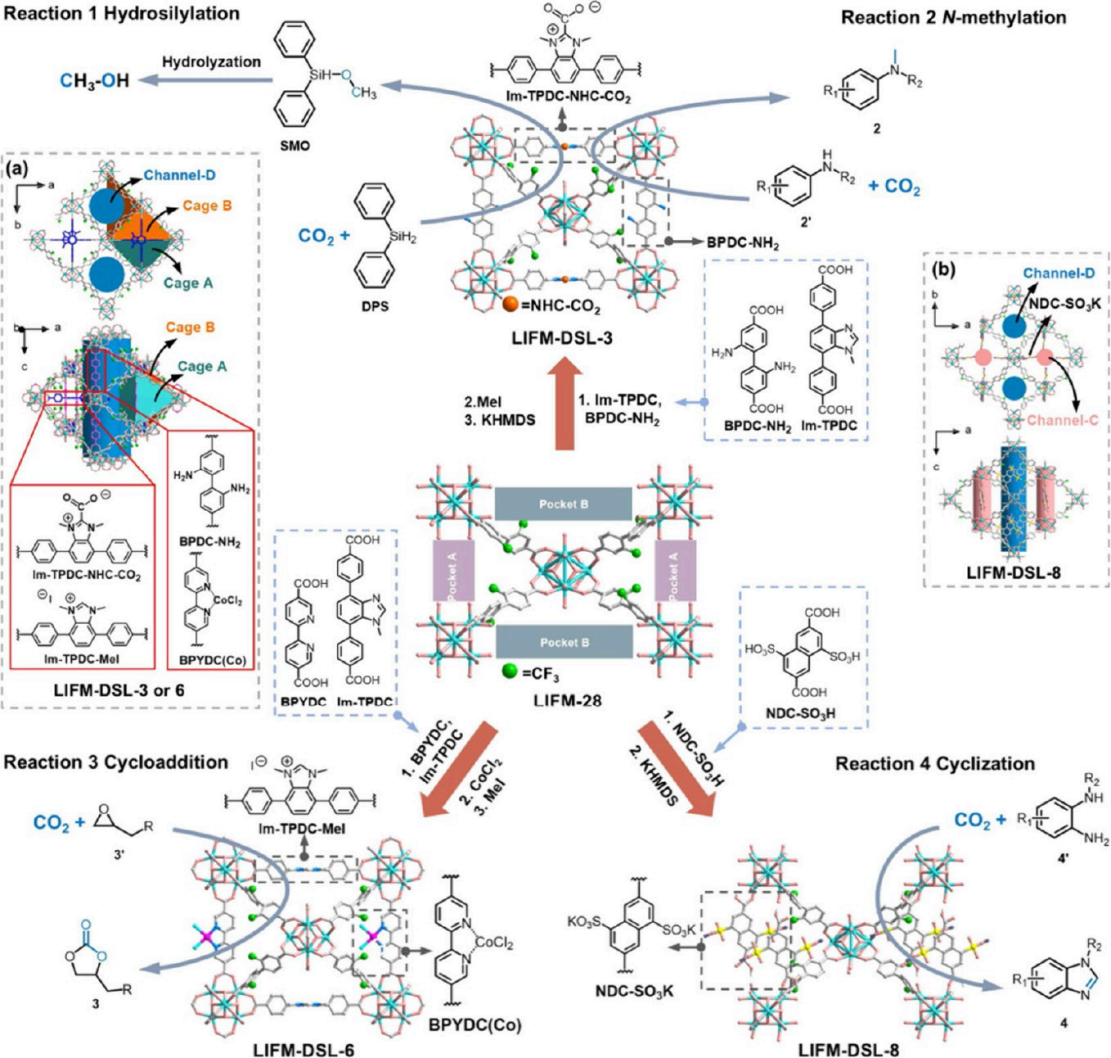
Illustration of multiple catalytic transformation of CO_2_ by catalytically active linkers installed LIFM-DSL frameworks. Reproduced with permission from ref 167. Copyright 2025, American Chemical Society.

Compared with physically encapsulated guests, the linker installation presents a uniquely powerful strategy for luminescent sensing by fixing the reporter molecules and recognition units at crystallographically defined positions inside MOF pores, thus controlling precisely the analyte binding, signal transduction, and framework stability.

Han et al. showed that linker modification can be used to achieve homochiral luminescent MOFs for enantioselective sensing by incorporating a d-camphorate chiral linker into the free coordination sites of PCN-700 (Figure 11a).^168^ The resulting MOF contains a crystallographically defined homochiral cavity that selectively binds enantiomeric substances like quinine and quinidine, resulting in differences in luminescence quenching efficiency with high selectivity factors (K_SV_(R)/K_SV_(S) ≈ 1.45–1.50), which allows quantitative analysis of enantiomeric excess based on luminescence intensity differences. The MOF structure exhibits high stability and recyclability, which confirms that linker modification is a precise method of performing chiral recognition and enantioselective luminescence sensing. Furthermore, Han et al. extended this finding by demonstrating that linker loading is suitable for designing quantitative redox-responsive luminescence turn-on sensors by incorporating redox-active dyes (resazurin, Amplex red, and resorufin) into the absent linker sites of PCN-700 (Figure 11b).^169^ The redox reactions can transform the loaded dyes into strongly emissive resorufin, resulting in visible coloration and intense fluorescence turn-on within 1 min, allowing micromolar detection of general oxidants and reductants in an aqueous environment. The lack of leaching and pH stability of the sensors is attributed to the two-ended binding of dyes, which outperforms guest-encapsulated sensing platforms.

**Figure 11:**
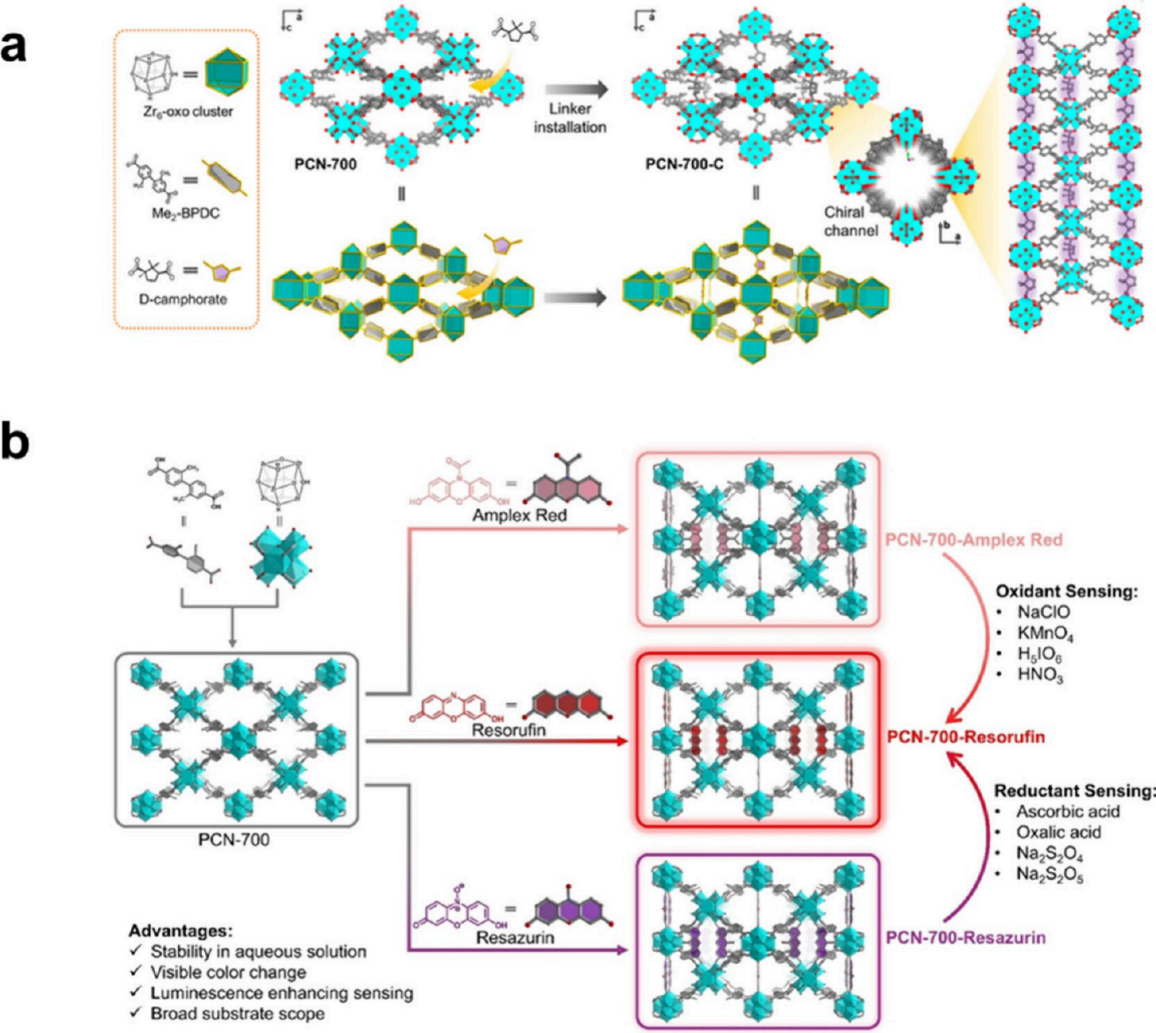
Illustration of (a) chiral linker installation in PCN-700 for enantioselective sensing. Reproduced with permission from ref 168. CC BY 4.0. (b) Redox-active dye installation in PCN-700 for redox-species sensing. Reproduced with permission from ref 169. Copyright 2025, Wiley-VCH.

#### 2.3.3 Pore Partitioning

The pore space partition (PSP) is a highly effective approach for enhancing gas separation and small molecule capture by dividing large pore cavities into multiple small pockets, as proposed by Bu and Feng’s research group.^170,171^ In 2015, Zhao et al. reported a symmetry-matching regulated ligand insertion strategy to achieve precise pore partitioning.^172^ They introduced a tripyridyl-type ligand into the MIL-88 frameworks to obtain a family of Ni-trimer-based MOFs, CPM-33 family, creating smaller pore sizes and showing superior CO_2_ uptake capacity. Zhou group demonstrated the postsynthetic installation of both organic linkers and metal clusters into the multicomponent MOFs guided by a kinetic analysis method. In this work, multiple organic linkers and metal ions/clusters were subsequently installed into the cavity of PCN-224 prototype, for example, in the retrosynthesis of PCN-202­(Ni)-Hf (Figure 12). After the pore partitioning, significantly enhanced H_2_ adsorption capacity in PCN-202­(Ni)-Zr has been observed.^173^

**Figure 12:**
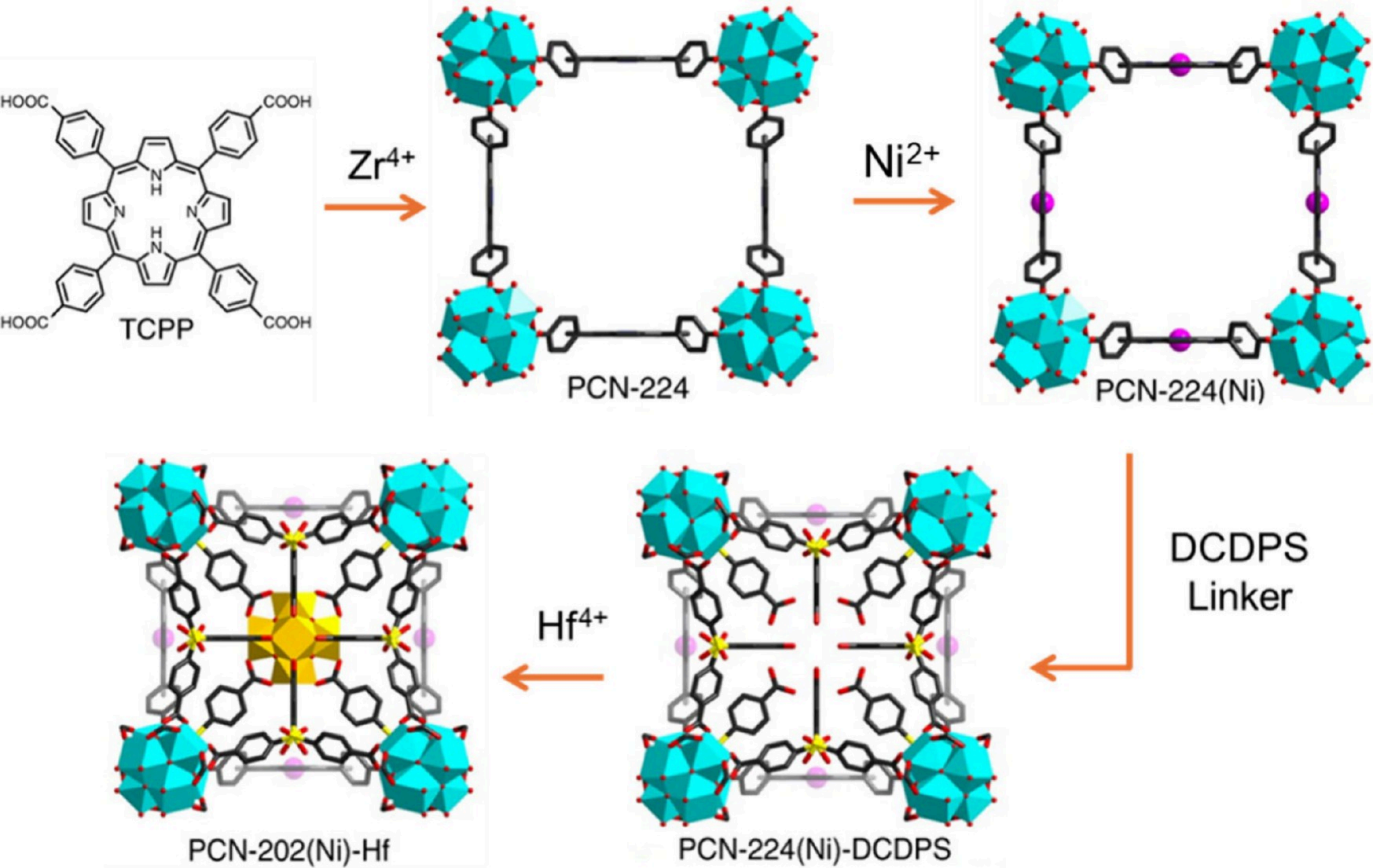
Retrosynthesis of PCN-202­(Ni)-Hf. Reproduced with permission from ref 173. CC BY 4.0..

#### 2.3.4 Post-Synthetic Functionalization (PSF) of Pore Surfaces

Postsynthetic functionalization (PSF) is one of the most widely investigated strategies for introducing functional groups onto pore surfaces while preserving the lattice structure.^174^ This approach enables the construction of novel materials with tailored properties for broadly applicable uses. As illustrated in Figure 13, pore-surface functionalization is typically categorized into three strategies. The first and most established method involves covalent modification of organic linkers, utilizing reagents to form covalent bonds with the existing framework linkers (Figure 13a). To functionalize the pore, the existing linkers in the parent MOFs must contain reactive functional groups such as amine or aldehyde. These sites can undergo a broad range of PSF reactions, including amide coupling,^90,175–177^ alkylation,^178,179^ imine condensation,^180,181^ “click” chemistry,^86,182,183^ and ring-opening reactions.^184,185^ The use of NH_2_–BDC ligand analogs as linkers, for example, has been widely observed in various MOFs. In 2010, Cohen group successfully transformed amine-containing MOFs, IRMOF-3 and MIL-53­(Al)-NH_2_, to amide-functionalized MOFs by introducing hydrophobic alkyl chains via PSM, thereby imparting hydrophobicity and improving moisture resistance in water-sensitive MOFs.^90^ More recently, Queen and co-workers reported a two-step postsynthetically grafting alkylamines into IRMOF-3 for enhanced CO_2_ adsorption.^186^ The amine groups of the NH_2_-BDC ligand underwent nucleophilic substitution with bromoacetyl bromide, producing acetyl bromide product, which then reacted with polyamines in a second reaction.

**Figure 13:**
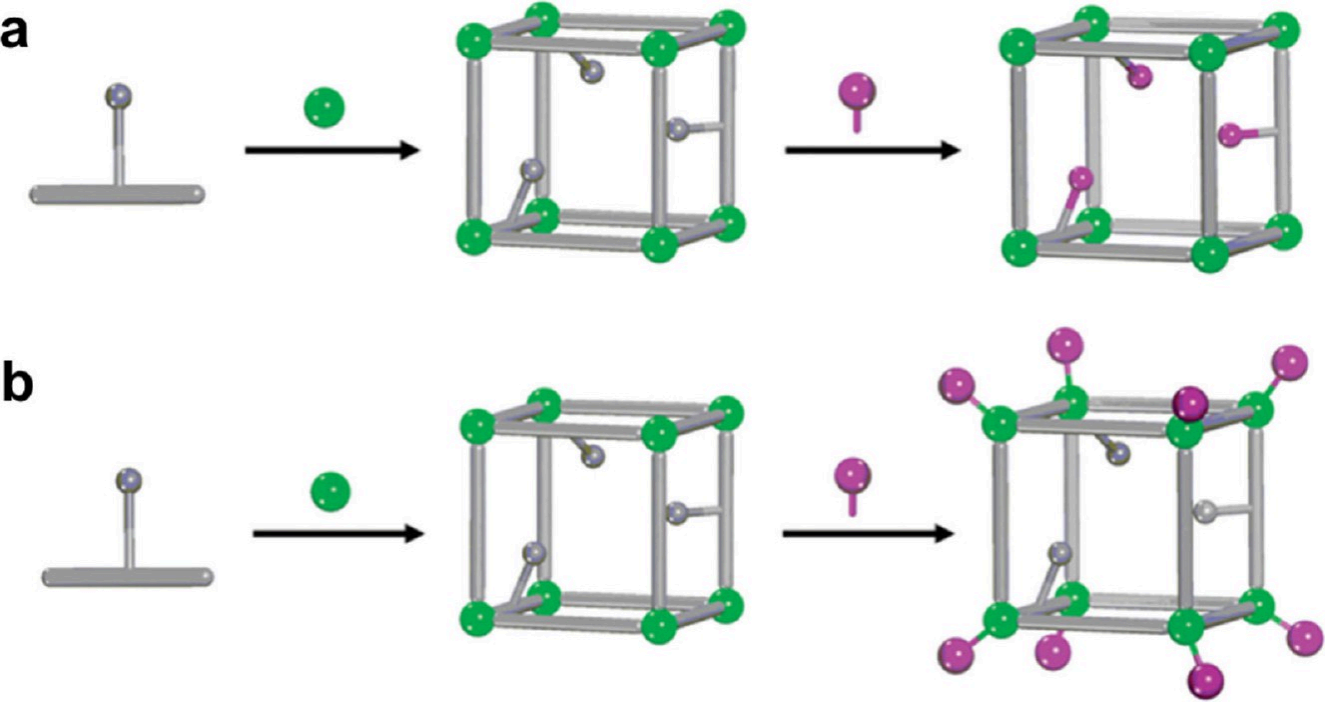
Postsynthetic functionalization (PSF) strategies, including (a) covalent modification of organic linkers and (b) coordinate covalent modification at the open metal sites (OMS). Reproduced with permission from ref 174. Copyright 2012, American Chemical Society.

Alternatively, coordinate PSF can be employed to functionalize the framework at the open metal site (OMS) or open coordination sites (OCS), where partially coordinated metal ions/cluster nodes or vacant Lewis acid sites are present (Figure 13b).^187^ These sites can facilitate framework functionalization through the introduction of different linkers, such as Lewis bases (e.g., amines, pyridines, or phosphines), small anions, or neutral ligands. By grafting these functional groups, the functionalized MOFs can be precisely tuned while preserving the crystallinity and porosity of the parent framework. For instance, Kim and co-workers first reported functionalized MIL-101­(Cr) by introducing chiral pyridyl modified organocatalytic l-proline-based ligands at the open Cr^3+^ sites, yielding chiral MOFs with outstanding catalytic activities in asymmetric aldol reactions.^188^ In another example, Liu et al. demonstrated the grafting of pyridyl-salicylimine (Py-SI) onto the open metal site of Cu-BDC MOF to obtain Cu-BDC/Py-SI. This material was subsequently used to coordinate with Pd^2+^ ion, yielding Pd@Cu-BDC/Py-SI MOF that can be used as a catalyst for Suzuki coupling.^189^

#### 2.3.5 Pore Expansion and Contraction via Guest-Induced Dynamics

Besides rigid structures, the postsynthetic method (PSM) serves as a tool for pore engineering with reversible structural transitions, thereby significantly altering the properties of MOFs. In this approach, functionalized linkers are introduced to induce guest-induced dynamics or internal framework interactions. Typically, pore expansion is triggered by the presence of external guests or other stimuli, whereas pore contraction occurs upon guest removal.^96^ For instance, in 2009, the Cohen group first reported on the use of chemical functionalization to modulate the breathing (guest-induced) effect in a MOF.^190^ By varying the lengths of alkyl side chains through PSM of NH_2_-bearing DMOF-1, they found that distinct substituents on functionalized linkers significantly impact structural flexibility and porosity.

#### 2.3.6 Hierarchical and Hybrid Strategies for Hierarchical Pore Creation

Recently, the construction of hierarchical pore structures, ranging from micro- to meso- and even macro-pores within MOF structure (HP-MOFs) has received special attention in the field of pore engineering, given the limitations of traditional microporous MOF pore sizes, which are too small (<2 nm) for targeted applications.^191^ Researchers have successfully developed several synthetic strategies for hierarchical MOF systems by introducing mesoporosity and functionalities/properties, such as modulated synthesis, soft/hard template method, stepwise ligand exchange, chemical etching, linker labilization, linker thermolysis, etc.^192,193^

Modulated synthesis has emerged as a widely used strategy to regulate the crystallization of HP-MOFs by introducing modulators during synthesis, thereby influencing the kinetics of nucleation and crystal growth. In 2011, Choi et al. first successfully introduced meso- and macropores into microporous MOF-5 crystal by varying the amount of 4-(dodecyloxy)­benzoic acid (DBA) as a modulator, achieving sponge- and pomegranate-MOF-5 without losing their crystalline nature.^198^ Later, Jiang et al. demonstrated a modulator-induced defect-formation approach to produce hierarchical porosity in the Zr-based UiO-66 (Figure 14a).^194^ By employing an excess of monocarboxylic acid modulators first coordinated with the Zr-oxo cluster, then partially replaced with a higher acidity carboxylic acid ligand. Finally, the modulator was removed, yielding HP-UiO-66 with high stability and optimized pore accessibility.

**Figure 14:**
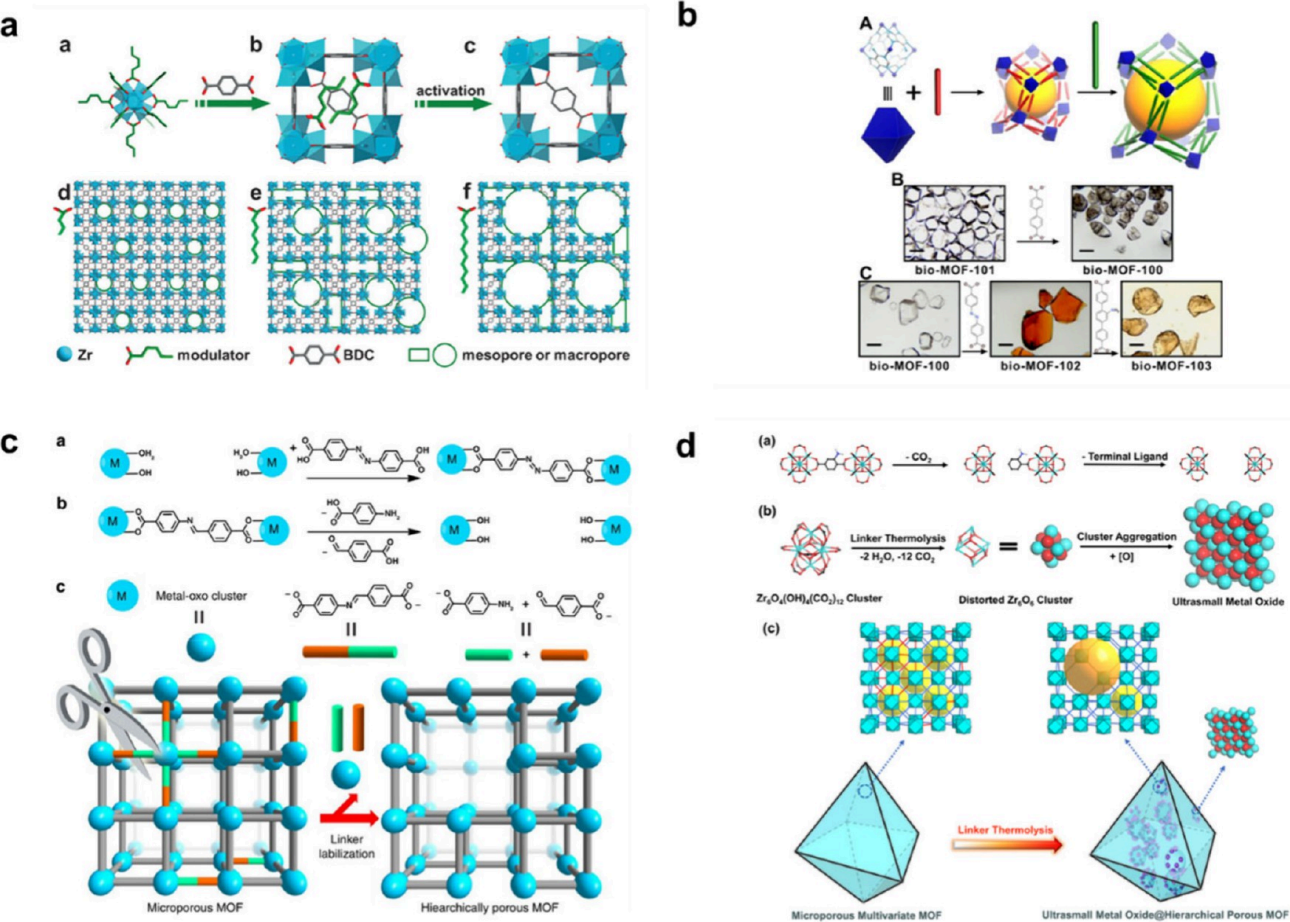
Some representative synthetic strategies for creating hierarchical MOFs, including (a) Modulator-induced defect-formation. Reproduced with permission from ref 194. Copyright 2017, Wiley-VCH. (b) Stepwise ligand exchange method. Reproduced with permission from ref 195. Copyright 2013, American Chemical Society. (c) Linker labilization. Reproduced with permission from ref 196. CC BY 4.0. (d) Linker thermolysis. Reproduced with permission from ref 197. Copyright 2018, American Chemical Society.

Rosi and co-workers first reported a stepwise ligand-exchange strategy to systematically expand the pore dimensions of bio-MOF-100 analogues by introducing linear dicarboxylate linkers of varying lengths.^195^ Starting material bio-MOF-101, containing zinc-adeninate clusters linked with a short dicarboxylate linker 2,6-naphthalenedicarboxylate (NDC), was progressively substituted with a longer BPDC (4,4′-biphenyldicarboxylate) to produce bio-MOF-100. Thereafter, the exchange processes were repeated by replacing the original linkers with slightly longer ABDC (azobenzene-4,4′-dicarboxylate) and much longer NH_2_-TPDC (2′-amino-1,1′:4,1′-terphenyl-4,4″-dicarboxylate) ligands, yielding distinct MOF products with progressively larger pore sizes and retained crystallinity (Figure 14b).

Kim and colleagues^199^ used a conventional chemical etching strategy to engineer hierarchical porosity in a microporous POST-66­(Y) MOF using water as the etchant, inspiring the widespread development of this method by investigating various etchants. However, this method is hindered by the limited applicability of MOFs and the lack of precise tunability of the framework. Inspired by this, the Zhou group presented, for the first time, the concept of linker labilization through a postsynthetic method to precisely control pore size and enhance porosity in microporous MOFs. In 2017, they demonstrated hierarchically porous PCN-160 via a two-step linker-labilization strategy that combined ligand exchange and etching.^196^ Initially, an acid-labile linker 4-carboxybenzylidene-4-aminobenzoate (CBAB) was exchanged into a stable PCN-160 framework bridged by a stable linker azobenzene-4,4′-dicarboxylate (AZDC). Due to the geometric similarity of the linkers, the framework integrity was maintained until the acid-labile CBAB linkers were selectively removed at a final hydrolysis step using acetic acid etching, producing hierarchically porous PCN-160 with excellent stability after acid treatment (Figure 14c).

Last but not least, Zhou and co-workers further developed a linker thermolysis strategy by incorporating both an ordinary linker and a thermolabile (amino-functionalized) linker into microporous multivariate MOFs.^197^ As illustrated in Figure 14d, the thermolabile linkers underwent selective decarboxylation process at relatively low temperature, leading to their removal from the framework and generating hierarchical mesopores with remaining crystallinity or structural stability. By adjusting the percentage of the thermal-sensitive linker, the size and distribution of the resulting hierarchical pores can be precisely controlled.

#### 2.3.7 MOF-on-MOF or Core-Shell Architectures

MOF-based hybrids have been extensively studied, due to their intrinsic merits, by the incorporation of two or more MOF units. Rapid developments have been achieved in this field since Kitagawa’s group first reported the synthesis of core–shell structured Cu-based MOFs on a substrate of Zn counterpart via an epitaxial growth strategy in 2009.^200^ However, this method primarily requires similar crystallographic parameters (lattice matching), which limits its applicability as most MOFs have distinct crystallographic parameters. To date, various synthetic strategies for core–shell structured MOF-on-MOF hybrids have been developed, including surfactant-assisted growth, heteroepitaxial growth, ligand/metal ion exchange, and nucleation kinetic guided growth.^201^ For instance, to overcome the limitation of the conventional approach, Zhou and coauthors developed a one-pot synthesis of a hybrid core–shell MOF (PCN-222@Zr-BPDC) with mismatched lattices guided by different nucleation kinetics between the core and shell MOFs (Figure 15a).^202^ Nonetheless, the versatility still limits this one-pot nucleation kinetic control strategy to generate clusters with different metal species. Later, Feng et al. reported the construction of a multifunctional hierarchical MOF-on-MOF with mismatched lattices via retrosynthetic design, with surface functionalization and retrosynthetic stability considerations (Figure 15b).^203^ This approach allows the controllable distribution of both functional groups and metal clusters within a framework architecture.

**Figure 15:**
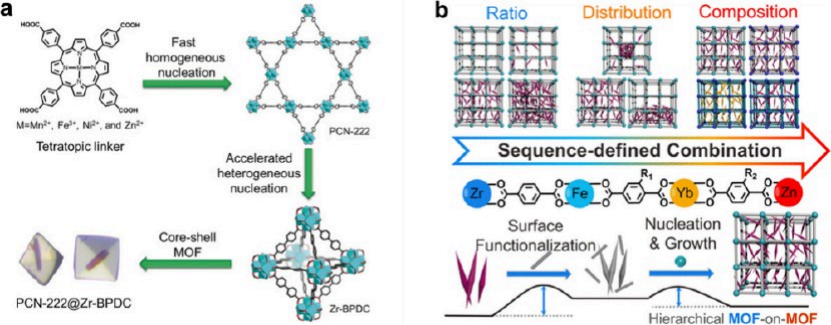
(a) Illustration of the kinetically guided synthesis of a hybrid core–shell PCN-222@Zr-BPDC. Reproduced with permission from ref 202. Copyright 2018, Wiley-VCH. (b) Formation of multivariate hierarchical MOF-on-MOF under two principles: surface functionalization and retrosynthetic stability considerations. Reproduced with permission from ref 203. CC BY 4.0.

## 4 Challenges and Outlook in Pore Engineering

Despite the significant progress that has been achieved in pore engineering, several fundamental issues still exist that must be addressed for the efficient and reliable programming and reprogramming of the pore structures of MOFs. Achieving quantitative and spatially uniform pore modification, especially for large crystals, remains challenging on account of diffusion-controlled rates, kinetic trapping, and site heterogeneity. Most of the current postsynthetic engineering toolkits are also only applicable for robust MOFs. Moreover, mapping local pore chemistry properties with macroscopic behavior is not a simple task because there is a lack of operando facilities that are sufficiently sensitive to probe site-selective adsorption, diffusion, and reactivity at the molecular scale inside confined geometries. The future of MOF-based research would depend on the synergy of single-crystal analysis, in situ analysis, and data-modeling tools that would establish rigorous structure–function correlations. Future techniques involving sequential linker chemistry, programmed deconstruction reactions, and adaptive guest chemistry are on the verge of developing a paradigm where MOFs would permit molecular-scale rewriting of their pore chemistry, paving the way for dynamically responsive materials that undertake complex multifunctionality not possible for traditional porous solids.

## 5 Conclusion

The development of redefined or refined pore structures in MOFs has transitioned from a static design philosophy to a dynamic, chemistry-oriented field, which allows for the deliberate design, control, and reprogramming of the pore systems in MOFs over multiple scales. With the integration of presynthetic design approaches, in situ control of self-assembly, and postsynthetic methods such as linker exchange, installation, and control over deconstruction, pore reprogramming in MOFs has advanced to allow for the independent control of pore size, topology, and functional microenvironments while maintaining crystallinity. These developments have caused pore reprogramming in MOFs to transform into dynamic and reprogrammable building blocks for separations, catalysis, sensors, and space-spanned chemistry, in which function is obtained from reprogrammed pore architectures. Further advances in synthetic control are envisioned to persist, leading to the development of optimized porous materials after crystallization, opening a pathway to fully programmable multifunctional framework solids.
